# Who should receive single-fraction palliative radiotherapy for gastric cancer bleeding?: An exploratory analysis of a multicenter prospective observational study (JROSG 17-3)

**DOI:** 10.1016/j.ctro.2023.100657

**Published:** 2023-07-03

**Authors:** Shuhei Sekii, Tetsuo Saito, Takashi Kosugi, Naoki Nakamura, Hitoshi Wada, Ayako Tonari, Hirofumi Ogawa, Norio Mitsuhashi, Kazunari Yamada, Takeo Takahashi, Kei Ito, Terufumi Kawamoto, Norio Araki, Miwako Nozaki, Joichi Heianna, Kenta Murotani, Yasuhiro Hirano, Atai Satoh, Tsuyoshi Onoe, Naoto Shikama

**Affiliations:** a Department of Radiation Oncology, Kita-Harima Medical Center, Hyogo, Japan; b Department of Radiation Oncology, Hyogo Cancer Center, Hyogo, Japan; c Department of Radiation Oncology, Osaka Police Hospital, Osaka, Japan; d Department of Radiation Oncology, Arao Municipal Hospital, Kumamoto, Japan; e Department of Radiation Oncology, Fujieda Municipal General Hospital, Shizuoka, Japan; f Department of Radiation Oncology, St. Marianna University School of Medicine, Kanagawa, Japan; g Department of Radiation Oncology, Southern TOHOKU Proton Therapy Center, Fukushima, Japan; h Department of Radiation Oncology, Kyorin University Hospital, Tokyo, Japan; i Division of Radiation Therapy, Shizuoka Cancer Center, Shizuoka, Japan; j Radiation Therapy Center, Hitachi, Ltd., Hitachinaka General Hospital, Ibaraki, Japan; k Department of Radiation Oncology, Seirei Mikatahara General Hospital, Shizuoka, Japan; l Department of Radiation Oncology, Saitama Medical Center, Saitama Medical University, Saitama, Japan; m Division of Radiation Oncology, Department of Radiology, Tokyo Metropolitan Cancer and Infectious Diseases Center Komagome Hospital, Tokyo, Japan; n Department of Radiation Oncology, Department of Radiology, Juntendo University School of Medicine, Tokyo, Japan; o Department of Radiation Oncology, National Hospital Organization Kyoto Medical Center, Kyoto, Japan; p Department of Radiation Oncology, Saitama Medical Center, Dokkyo Medical University, Saitama, Japan; q Department of Radiology, Nanbu Tokushukai Hospital, Okinawa, Japan; r Biostatistics Center, Kurume University, Fukuoka, Japan; s Department of Surgery, Southern Tohoku General Hospital, Fukushima, Japan

**Keywords:** Gastric cancer, Radiotherapy, Palliative treatment

## Abstract

**Purpose:**

Although the Palliative Prognostic Index (PPI) has been used to predict survival in various cancers, to our knowledge, no study has examined its applicability in gastric cancer. This study aimed to determine the baseline PPI cutoff value for recommending single-fraction radiotherapy in patients with bleeding gastric cancer.

**Materials and methods:**

This was a secondary analysis of the Japanese Radiation Oncology Study Group (JROSG) 17–3, a multicenter prospective study of palliative radiotherapy for bleeding gastric cancer. Discrimination was evaluated using a time-dependent receiver operating characteristic curve, and the optimal cutoff value was determined using the Youden index. A calibration plot was used to assess the agreement between predicted and observed survival.

**Results:**

We enrolled 55 patients in JROSG 17–3. The respective median survival times were 6.7, 2.8, and 1.0 months (p = 0.021) for patients with baseline PPI scores of ≤ 2, 2 < PPI ≤ 4, and PPI > 4. The areas under the curve for predicting death within 2, 3, 4, and 5 months were 0.813, 0.787, 0.775, and 0.721, respectively. The negative predictive value was highest when survival < 2 months was predicted and the Youden index was highest when the cutoff PPI value was 2. The calibration curve showed a reasonable agreement between the predicted and observed survival.

**Conclusion:**

Baseline PPI is useful for estimating short-term prognosis in patients treated with palliative radiotherapy for gastric cancer bleeding. A cutoff PPI value of 2 for estimating survival ≤ 2 months should be used to recommend single-fraction radiotherapy.

## Highlights

• PPI predicts < 2-month survival with palliative radiotherapy for gastric cancer bleeding.
• Single-fraction 8-Gy radiotherapy is recommended for patients with PPI > 2.
• Re-irradiation for rebleeding to be considered for longer survival than predicted.

## 1 Introduction

In patients with advanced gastric cancer, hemorrhage, discomfort, anorexia, pain and obstructive manifestations are of paramount concern. Despite advances in systemic therapies, the prognosis of these patients remains poor [1], with many requiring palliative care. Palliative radiotherapy is a treatment option for patients with hemorrhagic gastric cancer that is not amenable to surgery or chemotherapy.

The Japanese Radiation Oncology Study Group (JROSG) study 17–3 (UMIN-CTR: UMIN000029580), which is the first multicenter prospective study on palliative radiotherapy for gastric cancer, demonstrated a high bleeding response rate [2]. A secondary analysis of the JROSG 17–3 study investigating the temporal changes in symptom scores indicated that after radiotherapy, amelioration of dyspnea, pain, and stress was observed in all 55 patients after radiotherapy [3]. We found that while single-fraction radiotherapy resulted in a significant improvement in fatigue and stress over 2 months, multiple-fraction radiotherapy did not [3]. Multiple-fraction radiotherapy may have hampered the improvement in fatigue and distress owing to its toxicity or treatment burden; single-fraction radiotherapy should be a reasonable choice for patients with expected survival of ≤ 2 months.

Prognosis prediction is important for the management of patients with advanced cancer. The Palliative Prognostic Index (PPI) is a tool developed to predict the survival of patients with advanced cancer [4]. PPI is a straightforward, easy-to-use tool that considers factors such as performance status (PS), oral intake, edema, dyspnea at rest, and delirium. Historically, PPI cutoff values of 2 or 4 have been employed to predict the short-term survival of various cancer patients [4], [5]. Notwithstanding the abundance of studies on prognosis prediction in patients with various advanced cancer [6], [7], [8], [9], [10], to our knowledge, no study has specifically investigated prognosis prediction in patients with advanced gastric cancer treated with palliative radiotherapy. This study aimed to investigate the prognostic value of PPI in patients with bleeding gastric cancer and to study the selection of patients for single-fraction irradiation.

## 2 Materials and methods

The JROSG 17–3 study has been previously described in detail [2]. The primary inclusion criteria were patients who had received blood transfusions or exhibited hemoglobin levels < 8.0 g/dL. Participants who had undergone or were planned to undergo chemotherapy or molecular-targeted therapy within 2 weeks prior to and after the planned start date of radiotherapy were excluded from the study. At enrollment, PPI was evaluated along with sex, age, PS, stage, date and amount of blood transfusions, previous treatment, and comorbidities. The target volume and the dose fractionation were determined at the discretion of each radiation oncologist [4].

## 3 Statistical analysis

The study population was divided into three groups using historically used PPI cutoff values of 2 and 4 (i.e., ≤ 2, 2.5–4, and > 4) [4], [5]. The Kaplan–Meier method was employed to estimate the overall survival, which was defined as the time from enrollment until death from any cause. Patients who were lost to follow-up without experiencing death were censored on the last date they were known to be alive. The log-rank test was used to compare the overall survival among patients with different PPI values at baseline.

Model performance was evaluated from two aspects: discrimination and calibration. Discrimination performance refers to the ability of a model to distinguish between patients who will die earlier and those who will die later [11]. Discrimination was assessed using the time-dependent receiver operating characteristic (ROC) analysis [12]. The area under the curve (AUC) is the measure of discrimination, which takes a value between 0.5 and 1; a higher AUC value indicates superior model performance. Time-dependent cumulative sensitivity, dynamic specificity, positive predictive value (PPV), and negative predictive value (NPV) were estimated. A high PPV indicates that a high proportion of patients with a PPI > the cutoff value, i.e., a shorter predicted survival, actually die shortly. A high NPV indicates that a high proportion of patients with a PPI ≤ the cutoff value, i.e., a longer predicted survival, actually live longer. The Youden index, calculated as the sensitivity plus specificity minus 1, was used to identify the optimal cutoff value for PPI. The Youden index takes values between zero (a useless test) and one (a perfect test). The calibration performance describes the similarity between the probability values predicted using a model and the observed probabilities [11]. The agreement between the observed and predicted survival was assessed using a calibration curve.

Statistical analyses were performed using R version 3.6.2. The R package “timeROC” was used for the time-dependent ROC analysis.

## 4 Results

**Fig. 1:**
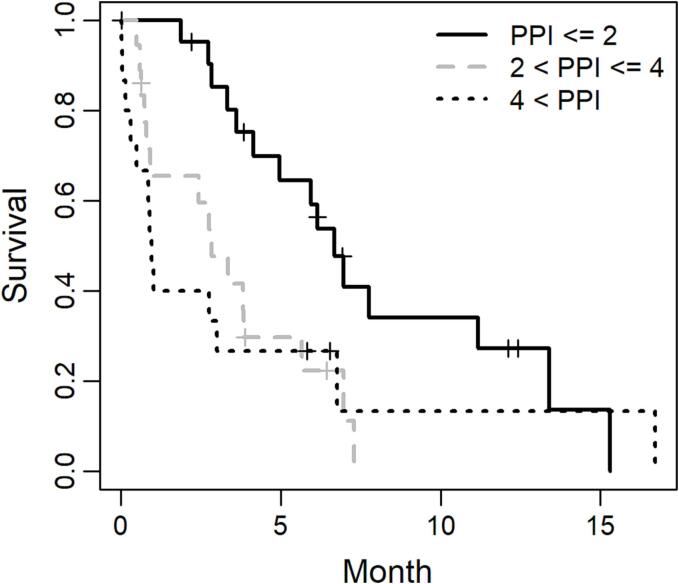
Kaplan-Meier curve of overall survival according to Palliative Prognostic Index.

A total of 55 patients were enrolled and analyzed in the JROSG 17–3 study, of whom 54 had primary gastric cancer and one had postoperative recurrent gastric cancer. Two patients did not receive the planned radiotherapy; one because of patient refusal, and the other because of patient mortality before the initiation of radiotherapy. The patient and tumor demographics are presented in Table 1. The respective numbers of patients with PPI values of ≤ 2, 2.5– 4, and > 4 were 22, 18, and 15. A median of five fractions was administered for a median total irradiation dose of 20 Gy (range, 8–45 Gy), where a single irradiation regimen was only 8 Gy. The median follow-up period, estimated using the reverse Kaplan–Meier method [13], was 12.1 months (95% CI 6.4 months–not estimable). There were 43 observed deaths. The median overall survival was 3.8 months (95% CI 2.8–6.1 months). The median survival times for the groups with PPI values of ≤ 2, 2.5– 4, and > 4 were 6.7, 2.8, and 1.0 months, respectively (p = 0.021; Fig. 1).

**Table 1:** Patient and tumor demographics.

		Number	%
Age (Y)			
	Median	73	
	Range	50–93	
Sex			
	Male	40	72
	Female	15	27
ECOG PS			
	0	7	13
	1	16	29
	2	18	33
	3	14	25
PPI at baseline			
	0	9	16
	1	11	20
	2	2	3
	2.5	13	23
	3.5	5	9
	4.5	4	8
	5	6	11
	6	1	2
	9.5	1	2
	10	1	2
	10.5	1	2
	11	1	2
T stage			
	< T4	11	20
	T4	38	69
	TX	6	11
N stage			
	N0	8	15
	N ≥ 1	38	6
	NX	9	16
M stage			
	M0	13	24
	M1	42	76
Histopathology			
	Adenocarcinoma	52	95
	Others	3	5
Total radiation dose (n = 53)			
	Median, Gy	20	
	Range, Gy	8–45	
	<10	12	23
	10–20	17	32
	21–30	22	42
	>30	2	3

ECOG, Eastern Cooperative Oncology Group; PS, performance status; PPI, Palliative Prognostic Index.*The Union for International Cancer Control 8th edition.

**Fig. 2:**
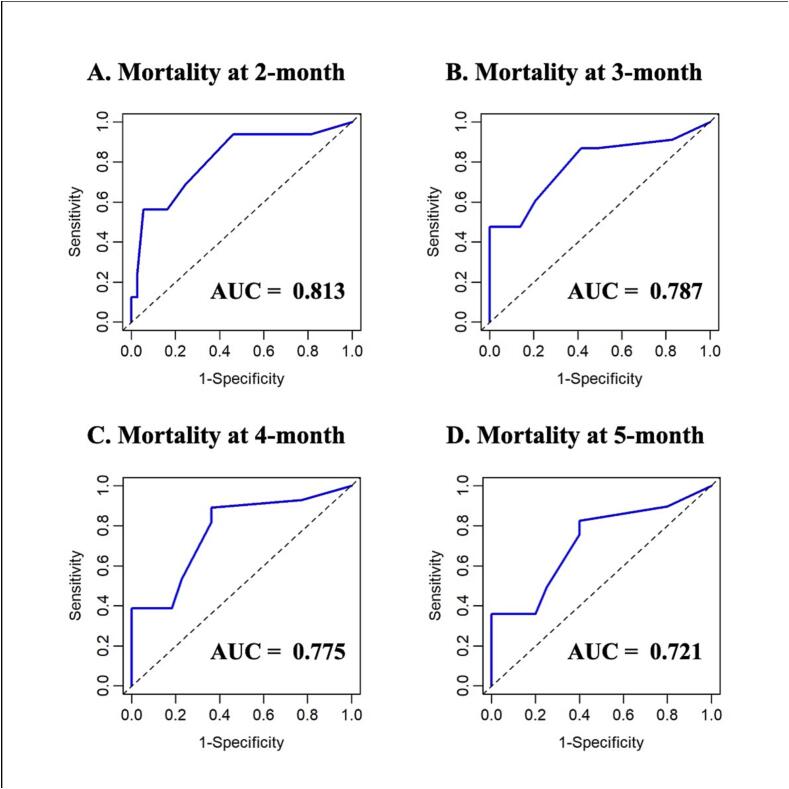
Time-dependent receiver operating characteristic curve for predicting death within 2 (A), 3 (B), 4 (C), and 5 months (D).

**Fig. 3:**
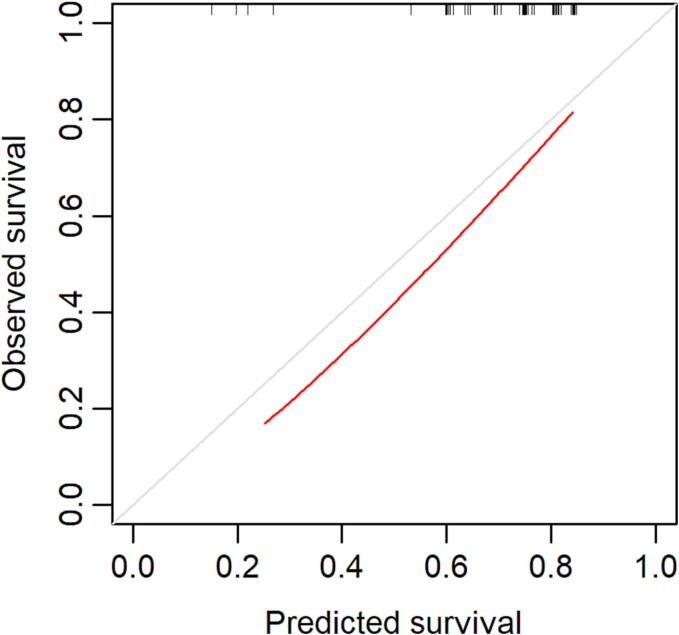
Calibration curve for the prediction of death within 2 months. Grey line represents perfect prediction. Calibration is good when the red curve is close to the grey line.

The AUCs for the prediction of death within 2, 3, 4, and 5 months from enrollment were 0.813, 0.787, 0.775, and 0.721, respectively (Fig. 2), demonstrating a gradual decrease in accuracy over the course of time. Table 2 displays the respective Youden index, time-dependent cumulative sensitivity, dynamic specificity, PPV, and NPV when using the PPI cutoff values of 1–5. For example, the respective PPV and NPV were 47% and 95%, when a cutoff value of PPI = 2 was used to predict death within 2 months. This means that approximately half of the patients erroneously predicted to live short actually lived long and most of the patients predicted to live long actually lived long. The Youden index was highest when the PPI cutoff value was set at 2 for the prediction of death ≤ 2 and 3 months; whereas the Youden index was highest when the PPI cutoff value was set at 1 for the prediction of death ≤ 4 and 5 months (Table 2). The calibration curve showed a reasonable agreement between the predicted and observed survival (Fig. 3).

**Table 2:** Accuracy to predict death within 2–5 months using baseline PPI cutoff values of 1–5.

		Baseline PPI cutoff value				
		1	2	3	4	5
Youden index						
	2-month	0.42	0.48	0.44	0.40	0.22
	3-month	0.38	0.45	0.40	0.34	0.22
	4-month	0.53	0.45	0.30	0.21	0.18
	5-month	0.43	0.36	0.24	0.16	0.16
Sensitivity (%)						
	2-month	94	94	69	56	25
	3-month	87	87	61	48	22
	4-month	89	82	53	39	18
	5-month	83	76	49	36	16
Specificity (%)						
	2-month	49	54	76	84	97
	3-month	52	59	79	86	100
	4-month	64	64	77	82	100
	5-month	60	60	75	80	100
PPV (%)						
	2-month	44	47	55	60	80
	3-month	58	62	69	73	100
	4-month	74	72	73	71	100
	5-month	74	72	73	71	100
NPV (%)						
	2-month	95	95	85	82	75
	3-month	83	85	72	68	62
	4-month	84	75	59	54	52
	5-month	72	65	52	48	47

PPI, Palliative Prognostic Index; PPV, positive predictive value; NPV, negative predictive value.

## 5 Discussion

A PPI cutoff value of 2 for the prediction of death within 2 months is useful for identifying patients who would benefit from single-fraction 8-Gy radiotherapy. This finding is supported by the following four reasons: First, the time-dependent ROC analysis indicated that the AUC decreased as the time progressed from 2 to 5 months, suggesting that PPI had a high predictive ability for short-term mortality. Second, our previous research found that only single-fraction radiotherapy resulted in significant improvements in fatigue and stress over two months, in contrast to multiple-fraction therapy [3]. Therefore, patients with expected survival of ≤ 2 months should receive single-fraction therapy. Third, the Youden index, which is used to find the best trade-off between sensitivity and specificity, suggested that the optimal PPI cutoff value for the prediction of death in ≤ 2 months was 2 in the selection from the PPI values of 1–5. Finally, the highest NPV was observed when survival ≤ 2 months was predicted and when the cutoff PPI value of 2 was used, as shown in Table 2. A high NPV may likely prevent the scenario where patients who are wrongly predicted to survive > 2 months actually die in ≤ 2 months; thus, extended fractionation is an unnecessary burden. Overall, we recommend single-fraction 8-Gy radiotherapy for patients with PPI > 2. The high NPV came with a tradeoff of the low PPV; approximately half of the patients predicted to die within 2 months may survive for over 2 months. Re-irradiation should be offered when rebleeding occurs [14].

Our findings that the prognosis was significantly shorter in the higher PPI groups is in line with that of previous studies. Morita and colleagues, who assessed PPI in various types of cancers including lung, gastric, and colorectal cancer, reported that the median survival times for the groups with PPI values of ≤ 2, 2.5–4, and > 4 were 134, 89, and 23 days, respectively [4]. The PROGRAD study, where the PPI was tested exclusively in patients treated with palliative radiotherapy, revealed that the median survival times for the three groups with the same classification of PPI values were 120, 55, and 39 days, respectively [5]. Our study provides evidence for patients with gastric cancer treated with palliative radiotherapy.

Prognostication is an integral component of decision-making in oncology, particularly in determining treatment options and referring patients to hospice care. However, oncologists have been shown to overestimate prognosis [15], likely resulting in the overuse of fractionated irradiation in patients with short survival. This can lead to a waste of the patients’ limited time, more adverse events, worsening of quality of life, and failure to complete palliative radiotherapy. Therefore, it is important to accurately identify patients who should receive single-fraction radiotherapy.

Single-fraction palliative radiotherapy is advantageous owing to its convenience, cost-effectiveness, and a high effectiveness. Although single-fraction radiotherapy has been recommended for patients with uncomplicated bone metastases [16], [17], [18] and spinal cord compression with a limited survival [19], [20], its use for abdominopelvic malignancies including gastric cancer has not been sufficiently supported by evidence. Although single-fraction radiotherapy is recommended for these patients by expert opinion, especially those with short-term prognosis [21], [22], [23], more evidence is required to further support these recommendations. Our study provides a new evidence regarding palliative radiotherapy for gastric cancer, an area that had hitherto been under-researched.

One inherent strength of this study may be that during our original prospective observational study, we did not set stringent eligibility criteria, in order to include a real-world patient population. The survival and patient characteristics such as PS do not seem to be substantially different from those of past retrospective studies on unselected patients in daily practice [24], [25], [26].

Our study had some limitations. Only 43 deaths were observed, and the relatively small sample size may have limited the analysis. Another limitation was the exploratory nature of the study. Further future studies are necessary to investigate the prediction of survival in patients treated with palliative radiotherapy for gastric cancer.

## 6 Conclusions

This study suggests that baseline PPI is useful for estimating short-term prognosis in patients treated with palliative radiotherapy for gastric cancer. A patient with a PPI value > 2 may be a reasonable candidate for single-fraction 8-Gy radiotherapy. This cutoff PPI value of 2, which exhibited the highest NPV in the prediction of survival ≤ 2 months, would be adequate to prevent the scenario where patients who are wrongly predicted to survive > 2 months actually die in ≤ 2 months and thus extended fractionation is unnecessarily used. The low PPV associated with this cutoff PPI value suggests the importance of reirradiation for re-bleeding in patients with good prognosis.

## Funding

This study was partially supported by the Japan Agency for Medical Research and Development (AMED, 22ck0106745h0001).

## Declaration of Competing Interest

The authors declare that they have no known competing financial interests or personal relationships that could have appeared to influence the work reported in this paper.
